# Verification of SNPs Associated with Growth Traits in Two Populations of Farmed Atlantic Salmon

**DOI:** 10.3390/ijms17010005

**Published:** 2015-12-22

**Authors:** Hsin Y. Tsai, Alastair Hamilton, Derrick R. Guy, Alan E. Tinch, Steve C. Bishop, Ross D. Houston

**Affiliations:** 1 The Roslin Institute and Royal (Dick) School of Veterinary Studies, the University of Edinburgh, Midlothian EH25 9RG, UK; Stephen.Bishop@roslin.ed.ac.uk (S.C.B.); Ross.Houston@roslin.ed.ac.uk (R.D.H.); 2 Landcatch Natural Selection Ltd., 15 Beta Centre, Stirling University Innovation Park, Stirling FK9 4NF, UK; Alastair.Hamilton@hendrix-genetics.com (A.H.); Derrick.Guy@hendrix-genetics.com (D.R.G.); Alan.Tinch@hendrix-genetics.com (A.E.T.)

**Keywords:** Atlantic salmon, growth, GWAS, marker-assisted selection, MAS, SNP, *PCNT*, *MEP1A*

## Abstract

Understanding the relationship between genetic variants and traits of economic importance in aquaculture species is pertinent to selective breeding programmes. High-throughput sequencing technologies have enabled the discovery of large numbers of SNPs in Atlantic salmon, and high density SNP arrays now exist. A previous genome-wide association study (GWAS) using a high density SNP array (132K SNPs) has revealed the polygenic nature of early growth traits in salmon, but has also identified candidate SNPs showing suggestive associations with these traits. The aim of this study was to test the association of the candidate growth-associated SNPs in a separate population of farmed Atlantic salmon to verify their effects. Identifying SNP-trait associations in two populations provides evidence that the associations are true and robust. Using a large cohort (*N* = 1152), we successfully genotyped eight candidate SNPs from the previous GWAS, two of which were significantly associated with several growth and fillet traits measured at harvest. The genes proximal to these SNPs were identified by alignment to the salmon reference genome and are discussed in the context of their potential role in underpinning genetic variation in salmon growth.

## 1. Introduction

The Food and Agriculture Organization (FAO) reported that the worldwide production of farmed finfish was approximately 66.6 million tonnes from 2011 to 2012, an increase of 26% compared with 2008 to 2009 [1]. The demands for high quality animal proteins are continuously expanding due to global economic development and human population increase. Aquaculture has a major role in fulfilling the increased requirement of protein consumption, and the continuous improvement of farming scale, sustainability and efficiency is required. Selective breeding for key production traits (such as feed efficiency and disease resistance) in finfish and shellfish species is an essential component of this improvement. However, aquaculture breeding schemes are generally fewer and less developed than terrestrial livestock and plants [2,3]. Gjedrem *et al.* [4] indicated that less than 10% of aquaculture production was based on genetically-improved stock. Notably, the annual genetic gain in selective breeding programmes of aquaculture species is typically higher than that of farmed terrestrial species [4], highlighting that genetic improvement of the key economic traits can be readily achieved.

The development of high throughput sequencing technologies has expedited the discovery of millions of genome-wide SNPs, particularly for salmonid species, which have high economic values; e.g., Atlantic salmon [5,6], rainbow trout [7,8] and sockeye salmon [9]. To date, for Atlantic salmon, traits, such as fillet colour, sexual maturation and fat percentage, have been initially studied using genome-wide association (GWA) analyses using an SNP array with approximately 6 K markers [10,11]. Additionally, GWAS for host resistance to sea lice [12], host resistance to *Piscirickettsia salmonis* [13] and early growth traits [14] have been performed using higher density SNP chips (50 or 132 K SNPs). Around 70 to 100 million years ago, the ancestor of modern salmonids underwent a whole genome duplication (WGD) event [15,16], which was followed by extensive modifications of both the genome and transcriptome and is still under the process of returning to diploidy [17,18]. The relics of the duplicated genomes generated by WGD complicate the discovery and interpretation of genomic variation, partly due to the difficulty in distinguishing true segregating polymorphism from paralogous variation [17]. Nonetheless, the vast majority of SNPs discovered to date in salmonid species segregate in a diploid manner [19].

The heritability of growth traits, such as body weight and length, in Atlantic salmon is moderate to high (e.g., [10,20,21]); but these complex traits are usually considered highly polygenic, and the underlying physiological basis for growth is likely to involve networks of many interacting genes. Typically, functional networks regulating growth-related traits involve hundreds of candidate genes [22,23]. Detecting and investigating the function of each individual gene within such complex networks is practically unfeasible. However, clues to the possible roles of particular candidate genes can be determined by associating genomic variation within or close to the gene with phenotypic variation in the trait of interest on a population scale. Herein lies the potential of GWAS to inform the underlying biology of the trait in question, in addition to providing potential markers for selective breeding programmes. Several previous studies of the association between candidate gene polymorphisms and phenotypic variation in salmon populations have focused on well-known candidates with previously-demonstrated physiological roles in the trait of interest (e.g., [19,24,25]). With the advent of high density and high throughput genotyping assays, GWAS and subsequent alignment to a reference genome [26] can identify positional candidate genes in a more systematic manner. However, with all association studies, it is important to assess the robustness of any putative significant result by testing the association between the SNP and the trait in a separate population/study. Therefore, the aims of this study were (i) to test the association of a subset of the most significant SNPs associated with weight and length of juvenile salmon [14] in another large cohort of fish and (ii) to identify and discuss putative candidate genes proximal to the SNPs that may directly contribute to variation in the growth phenotypes.

## 2. Results

### 2.1. Heritability Estimation

The population used in the analysis was a random subset of a larger population (Population 2) measured for overall and component weight traits, colour and fat content. Heritabilities of fillet-related traits were moderate to high (0.52 to 0.53), whereas the waste weights (e.g., head weight) were approximately 0.3. The heritability of fat percentage and fillet colour was slightly lower (0.14 to 0.18). The phenotypic and genetic correlations were high for all of the weight-related traits (*r*~0.96 to 0.99), but with little correlation between weight traits and fillet colour (*r* = −0.08). A summary of the heritability estimation and general statistics are given in Table 1, and they were consistent with estimates made on the larger population analysed previously [19].

**Table 1:** The summary statistics and heritability estimates for the harvest traits.

Traits	Mean (SD)	Heritability (SE)
Harvest weight (kg)	2.65 (0.72)	0.52 (0.05)
Head weight (kg)	0.30 (0.12)	0.21 (0.03)
Body waste weight (kg)	0.34 (0.15)	0.15 (0.02)
Total waste weight (kg)	0.67 (0.21)	0.32 (0.04)
Gutted weight (kg)	2.42 (0.65)	0.53 (0.05)
Deheaded weight (kg)	2.11 (0.57)	0.52 (0.05)
Fillet weight (kg)	1.76 (0.48)	0.53 (0.05)
Fat percentage (%)	13.2 (5.98)	0.18 (0.03)
Fillet colour (20–34)	28.9 (0.74)	0.14 (0.03)
Gut weight (kg)	0.22 (0.08)	0.30 (0.04)

### 2.2. Association between SNPs and Traits of Interest

**Figure 1:**
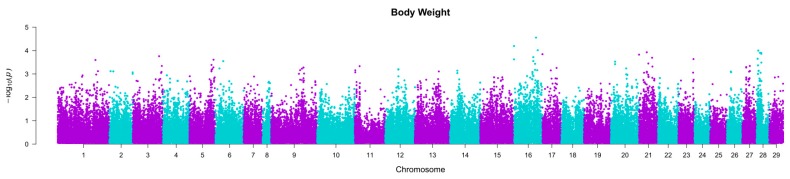
The Manhattan plot of body weight in the GWAS of Population 1 [14]. The Bonferroni genome-wide significance threshold is *p*~4.50 × 10^−7^.

**Figure 2:**
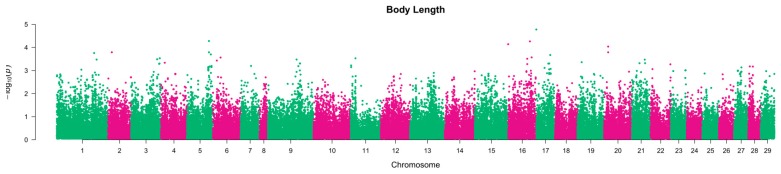
The Manhattan plot of body length in the GWAS of Population 1 [14]. The Bonferroni genome-wide significance threshold is *p*~4.50 × 10^−7^.

Based on the results of the 2007 year group (Population 1) GWA analysis, 16 nominally significant SNPs were selected for genotyping in the 1999 year group (Population 2). These SNPs were chosen from QTL regions on chromosomes 16, 21 and 28 for weight and chromosomes 5, 16, 17 and 20 for length (Figure 1 and Figure 2). Assays failed for six SNPs, and two more were monomorphic (details of selected markers were tabulated in Table S1). Of the remaining eight successfully genotyped SNPs, two were significantly associated with several growth traits (Table 2).

**Table 2:** Results of the association analysis including the predicted mean value (and standard error) and proportion of additive genetic variance due to SNP (PVE) for each trait and genotype class.

Traits	AX88141678 (Gene: *MEP1A*)				AX88270804 (Gene: *PCNT*)			
	A/A	A/G	G/G	PVE (%)	A/A	A/G	G/G	PVE (%)
# of fish	*n* = 651	*n* = 436	*n* = 52		*n* = 281	*n* = 581	*n* = 265	
Harvest weight	2.59 (0.04)	2.63 (0.05)	2.33 (0.1) **	0.3	2.66 (0.05)	2.60 (0.04)	2.50 (0.06) *	1
Head weight	0.30 (0.01)	0.30 (0.01)	0.26 (0.02) **	1.3	0.31 (0.01)	0.3 (0.01)	0.28 (0.01) **	1
Body waste weight	0.33 (0.01)	0.35 (0.01)	0.32 (0.02)	0.1	0.34 (0.01)	0.35 (0.01)	0.31 (0.01) **	3
Total waste weight	0.65 (0.01)	0.67 (0.01)	0.61 (0.03)	0	0.66 (0.02)	0.67 (0.01)	0.61 (0.02) **	2
Gutted weight	2.37 (0.04)	2.39 (0.04)	2.19 (0.09) *	0.2	2.41 (0.05)	2.38 (0.04)	2.28 (0.05) **	1
Deheaded weight	2.07 (0.03)	2.10 (0.03)	1.94 (0.08)	0.05	2.10 (0.04)	2.09 (0.03)	1.99 (0.04) *	1
Fillet weight	1.71 (0.03)	1.76 (0.03)	1.59 (0.07) **	0	1.76 (0.04)	1.72 (0.03)	1.67 (0.04)	1
Fat percentage	13.19 (0.27)	13.12 (0.31)	12.41 (0.84)	0.4	13.65 (0.39)	13.17 (0.28)	12.45 (0.4) *	4
Fillet colour	28.96 (0.04)	28.90 (0.05)	29.03 (0.12)	0.1	28.98 (0.06)	28.90 (0.04)	28.97 (0.06)	0.02
Gut weight	0.21 (0)	0.22 (0)	0.20 (0.01)	0.01	0.22 (0.01)	0.22 (0)	0.20 (0.01) **	3

* Overall SNP *p* < 0.1; ** overall SNP *p* < 0.05.

The SNP AX88270804 was significantly associated (*p* < 0.05) with most of the fillet and waste traits, including a suggestive association with fat content (*p* < 0.1). The adenine allele corresponds to higher trait means for the carcass weight and fatness traits. The SNP AX88141678 was associated with overall harvest weight, head weight and gutted weight (*p* < 0.05). At this SNP, the adenine allele was also associated with higher trait means for the carcass and overall weight traits. The estimation of the additive genetic variation explained by the SNPs indicated that AX88270804 explained a small percentage of the overall variation in fillet traits (~1%), waste traits (2% to 3%) and fat percentage (4%). The SNP AX88141678 explained approximately 1% of the additive genetic variation in the weight-related traits (Table 2). To account for variation in the overall size of the fish when analysing component traits, Model (1) was preformed, including harvest weight as a covariate. In this analysis, most of the SNP-trait associations were no longer significant, but SNP AX88270804 showed an association with body waste weight and total waste weight.

### 2.3. QTL Region Characterization and Putative Gene Identification

The corresponding flanking sequences for the two significant SNPs were aligned with the reference genome (assembly GCA_000233375.4), and the putative genes proximal to the SNPs were identified, indicating that the loci AX88141678 (chr. 5) and AX88270804 (chr. 16) were located within *MEP1A* (meprin A subunit beta-like) and *PCNT* (pericentrin), respectively. AX88270804 was located in an exon (non-synonymous), whereas AX88141678 was in a non-coding region (Table 2). The details of all SNPs tested in the current study are given in Table S1.

The main results of the GWA analysis in Population 1 are given in Tsai *et al*. [14]. However, due to the recent availability of a chromosome-anchored reference genome sequence assembly for Atlantic salmon (GCA_000233375.4), we used BlastN to align the flanking sequence of the SNPs on the array with the assembly to identify their putative chromosome and position. This information was used to draw Manhattan plots to view the QTL regions from which the candidate SNPs were chosen (Figure 1 and Figure 2).

## 3. Discussion

Abundant SNPs discovered by modern sequencing technologies and bioinformatics tools have allowed us to better understand the association between genomic variation and production traits in aquatic species [27]. In a recent study, we applied a high density SNP array (~132 K) [6] to identify candidate markers associated with weight and length traits in a farmed salmon population measured at one year of age [14]. To test a subset of promising SNPs from the previous study in a different population, we successfully genotyped eight SNPs in a population of 1152 salmon with growth and harvest-related traits measured at three years of age. Two SNPs were found to be significantly associated with several growth and harvest traits in the second population, implying that these SNPs are linked to QTL with effects on growth at multiple stages of the salmon production cycle. For the remaining six SNPs where no significant association was detected, this may reflect false positives in the initial study or false negatives in the current study. Alternatively, SNPs may have specific lifecycle stage-specific effects on growth that were not observed in both studies due to the difference in age at which the salmon were measured (one and three years respectively). While only weight and length were measured in the GWAS [14], there were eight fillet- and carcass-related traits measured in the current study. Therefore, for the two SNPs that were validated in the current study, the use of these additional measurements helps to determine a more specific growth phenotype associated with the SNP effects. For example, the SNP AX88270804 was associated with fat percentage in the current study, which indicates that the faster growth associated with the favourable allele also leads to increased fat content of the fish.

Alignment of the SNP flanking sequences with the Atlantic salmon reference genome predicted that AX88270804 was a synonymous exonic SNP within the *PCNT* gene and showed a significant association with several muscle and skeletal growth traits (*p* < 0.05) in Population 2 (current study) and growth traits (*p*~10^−4^) in Population 1 [14]. The SNP explained between 1% and 4% of the genetic variation in various harvest traits. In humans, the *PCNT* gene encodes the centrosome protein pericentrin, which contributes to the organisation of the mitotic spindle for the segregation of the chromosomes during cell division, thus influencing cell cycle progression. Mitotic centrosome dysfunction caused by pericentrin mutations can be expected to cause disturbances in cell division and is known to result in seriously stunted growth of the body and brain [28,29]. Interestingly, the SNP within the *PCNT* gene in salmon also has a suggestive association with fat percentage, explaining approximately 4% of the genetic variation. As expected, the allele associated with faster growth is also associated with increased fatness (Table 2). Major mutations in the *PCNT* gene in humans also affect adipocyte differentiation and can result in dyslipidemia as part of a wider insulin resistance syndrome. The fact that *PCNT* function is necessary for normal growth and lipid regulation in humans raises the possibility that further minor genetic variation within and around the gene may contribute to phenotypic variation in these traits. However, the role of the pericentrin in salmonid species has not yet been established.

The SNP AX88141678 was found in the intronic region of the *MEP1A* gene, which encodes meprin A subunit alpha. Meprins are zinc metalloendopeptidases that are predominantly found in kidney and intestinal brush border membranes in mammals and are known to play a role in protein metabolism [30]. Like *PCNT*, little is known about the function of *MEP1A* in Atlantic salmon, but interestingly, diet manipulation in another salmonid species (rainbow trout (*O. mykiss*)) has been shown to result in marked expression changes of *MEP1A* in the intestine [31]. In addition, *MEP1A* expression was shown to differ between domesticated and wild brook char (*Salvelinus fontinalis*) and its putative effect on growth factors was postulated to be the underlying mechanism for the higher expression in selected fish [32]. Therefore, while the association with growth traits may be due to variation in nearby candidate genes, the association of an SNP within the *MEP1A* gene and growth traits and its postulated functional connection to the growth traits raise the possibility that the causative effect underlying this association may be mediated via the *MEP1A* gene itself. It is worth noting that the genotype means for the SNP suggest an overdominance effect, which may explain why the additive variation explained is very small (Table 2).

Loci AX88141678 and AX88270804 were mapped to chr. 5 and chr. 16 using sire-based linkage mapping, respectively [6], and alignment with the reference genome assembly. A recent quantitative trait loci (QTL) mapping study by our group [20] in the same population as the current study showed that chr. 16 harbours loci affecting several growth traits with chromosome-wide significance in a sire-based analysis, although no QTL were detected on chr. 5. To date, there is a lack of consistency between the locations of the QTL affecting growth traits in different studies and commercial salmon populations [20,33,34,35]; therefore, the growth traits are considered to be regulated by population-specific and polygenic factors. Further, while the association between the *PCNT* and *MEP1A* candidate gene polymorphisms and growth-related phenotypes measured in two different populations of salmon is encouraging, the direction of the allelic effects between the two studies was generally not consistent (see Table S2). For both SNPs in the current study, fish carrying two copies of the adenine allele had better growth performance than other genotypes, whereas in Tsai *et al.* [14], this genotype was associated with lower weight and length values. This may be due to opposing effects in different lifecycles and environments (freshwater *versus* seawater). A genotype by environment interaction has been shown to be evident for the direction of association of individual SNPs (e.g., [36]). Alternatively, these SNPs may be marking QTL some distance away, and the relationship between marker and QTL may vary from population to population. The QTL regions identified in the GWAS cover a relatively large region of the chromosomes (Figure 1 and Figure 2). As such, while identifying chromosomal regions and putative genes harbouring variation contributing to growth phenotypes in salmon is of biological interest, it is unlikely that specific marker-assisted selection for these individual loci will be of high value, in particular for growth traits, which are directly measurable on the selection candidates themselves. This is particularly the case because genomic prediction using relatively few genome-wide markers can lead to very accurate prediction of breeding values for complex traits, such as growth (e.g., accuracy ~0.7 for juvenile weight and length in [14]). Therefore, genomic selection-based breeding schemes are likely to be increasingly utilised for the improvement of polygenic traits as genotyping technology becomes more affordable [14,37], especially for those traits with high economic value and that are difficult to be visualized (e.g., milk yield in dairy and fillet weight in fish).

## 4. Experimental Section

### 4.1. Animals

The GWAS used to identify the SNPs with putative association with growth in commercial salmon populations was based on the 2007 year group population of the Landcatch Natural Selection (LNS; Ormsary, UK) broodstock that were measured for weight and length at the end of the freshwater period (~1 year old; “Population 1”) [14]. To test the candidate SNPs in a new population, 1152 individuals were randomly selected from a larger population (*n*~5000) comprising the 1999 year group of LNS broodstock that were measured for weight and other fillet traits at harvest (“Population 2”). The 1152 genotyped fish were across 191 full sibling families from 131 sires and 185 dams. The phenotypes were measured by LNS at harvest (approximately 3 years old), including overall harvest weight (kg), gutted weight (kg), deheaded weight (kg), fillet weight (kg), head weight (kg), gut weight (kg), body waste weight (kg) and total waste weight (kg), fat percentage (% as estimated using a Torry Fatmeter (Distell Ltd., Aberdeen, Scotland)) and fillet colour (assessed visually using the Roche SalmoFan scale (Hoffmann-La Roche, West Sussex, UK), ranging from 20 (Yellow) to 34 (Red)). The body waste weight was calculated as deheaded weight minus fillet weight (weight of vertebrae and caudal fin), and total waste weight was by head weight plus body waste weight. Details of the population and phenotype measurement are given in Tsai *et al.* and Peñaloza *et al.* [20,25]. An adipose fin tissue sample of each individual was clipped and retained for DNA extraction using DNeasy-96 tissue DNA extraction kits (Qiagen, Crawley, UK).

All animals were reared in accordance with all relevant national and EU legislation concerning health and welfare. Landcatch is an accredited participant in the RSPCA (Royal Society for the Prevention of Cruelty to Animals) Freedom Foods standard, the Scottish Salmon Producers Organization Code of Good Practice and the EU Code-EFABAR (http://www.responsiblebreeding.eu/) Code of Good Practice for Farm Animal Breeding and Reproduction Organizations.

### 4.2. SNP Selection and Genotyping

The candidate SNPs were selected based on two relevant studies [14,20]. Firstly, a GWA analysis was performed in Population 1 to select the candidate markers for genotyping [14], and a proportion of the SNPs surpassing a nominal significance (*p*~10^−^^3^) were selected. Secondly, chromosome 20 was identified as containing loci affecting growth and fillet-related traits in Population 2 [20]. Therefore, two SNPs with nominally significant association with weight and length (*p*~10^−^^2^) [14] from this QTL region were also included in the shortlist for further investigation. The details of candidate SNPs are given in Table S1. In total, sixteen candidate SNPs were selected for assay design and genotyping in Population 2, of which eight were successfully genotyped and showed segregation. Candidate SNP markers and their flanking sequences were provided to LGC Genomics (Herts, UK) for the design of “kompetitive allele-specific PCR (KASP)” assays (see KASP technique details at [38]) for genotyping with 1152 offspring in Population 2.

### 4.3. Statistical Analysis

#### 4.3.1. Heritability Estimation and SNP Associations

The heritability of the traits was calculated as described previously [20]. The simple animal model (Model (1)) was used to estimate the additive genetic effect of each SNP genotype (G):

***Y =*μ *+ G + A + e*** (1)

where ***Y*** represents the observed phenotype, **μ** is the overall mean of the trait, ***G*** is the fixed effect of the SNP genotype, ***A*** is the additive genetic effect and ***e*** is the residual error. For estimating heritability, the equivalent model was used, but without the SNP effect (***G***) using the model:

*h^2^_a_ =* σ^2^_a_/σ^2^_p_ (2)

where σ^2^_a_ is the additive genetic variance and σ^2^_p_ is the total phenotypic variance. The analysis was performed by ASReml 3.0 [39].

#### 4.3.2. Allelic Substitution Assessment

The allelic substitution effects of informative SNPs were estimated using Model (1) performed by ASReml 3.0 [39]. The SNP genotype was fitted as the fixed effect in the analysis. The additive effect of the candidate marker was calculated as the difference of the predicted phenotypic means of two homozygotes divided by two, which was given as (AA − BB)/2, and the dominance effect was AB − ((AA + BB)/2), where the AB represents the predicted phenotypic means from heterozygote and AA or BB are from homozygote in the statistical analysis. The proportion of genetic variance due to SNP (PVE) was also estimated, by the following equation [40]:

PVE = [2*pq* (α + δ(*q* − *p*))^2^]/*V*_A_ (3)

where α and δ are the additive and dominance effect, respectively, *p* is the frequency of the most frequent allele, *q* is the frequency of the minor allele and *V*_A_ is the total additive genetic variance of the trait obtained when no SNP effects are included in the model.

### 4.4. Candidate Gene Identification

To identify candidate genes near the significant SNPs, the flanking sequence was aligned to the Atlantic salmon reference genome assembly (GCA_000233375.4), and the corresponding genome contig and position of the SNPs were noted. Approximately 20 kb of sequence surrounding the SNPs were repeat masked (retrieved from [41]), and a BlastX analysis was used to detect putative genes within the vicinity of the SNPs.

## 5. Conclusions

In genome-wide association studies of complex and polygenic traits, the significant SNPs identified are likely to contain a mix of true associations and false positives. Therefore, verification of GWAS findings in a separate population is an important validation step, and SNP associations identified in more than one population are more likely to be reflecting real QTL. We identified two (out of eight successfully genotyped) SNPs that showed an association with growth traits in two different populations, and two different lifecycle stages, in Atlantic salmon. The SNPs are within the pericentrin and meprin alpha genes, which both have potentially relevant functional connections to the growth and harvest traits studied. Further investigation of these candidate genes may be merited to identify putative causative variation.

## Supplementary Materials

Supplementary materials can be found at http://www.mdpi.com/1422-0067/17/1/5/s1.

## Abbreviations

GWAS: genome-wide association study

*MEP1A*: meprin A subunit beta-like

*PCNT*: pericentrin

QTL: quantitative trait loci

SNP: single nucleotide polymorphism
