# Supplementary material for: Verification of SNPs Associated with Growth Traits in Two Populations of Farmed Atlantic Salmon
Source: Int J Mol Sci. 2015 Dec 22;17(1):5. doi: 10.3390/ijms17010005 (PMC4730252; doi:10.3390/ijms17010005)
Supplement: Supplementary file 1 [file ijms-17-00005-s001.pdf]

Supplementary Materials: Verification of SNPs Associated with Growth Traits in Two Populations of Farmed Atlantic Salmon

Hsin Y. Tsai, Alastair Hamilton, Derrick R. Guy, Alan E. Tinch, Steve C. Bishop and Ross D. Houston

Supplementary Table 1. The markers selected from GWA analysis for verification.

Sources	Marker ID	*p*-value *	Marker and Flanking Sequence
Top 0.5% SNPs in growth traits	AX87956267	2.37×10^{-3} (L)/ 1.45×10^{-3} (W)	CCATGTAGTTTTTGCATTCCAAAAGATCTTGCTGC**M**AA GAGCTAATGATTATTACAGATATTATGTTATTT
	AX88290778	7.27×10^{-4} (L)/ 1.03×10^{-3} (W)	TGGCACTTTTCAAATATTGAATTGGAATTTCAATT**M**AT GTCCTGAATTGACCATGGCTCTGGATAGAAACT
	AX87963258	1.65×10^{-5} (L)/ 1.42×10^{-4} (W)	CCTGTATGAGGGCAGCGAATGCTGGGTTCTGGAGG**Y**TG GCATGAATACCTAGGAGAGAGAATGGTTGTATT
	AX88270804	1.32×10^{-3} (L)/ 6.96×10^{-4} (W)	TCAGAGGTCTCGCTGGCGTCTCGTGAGACAGAGAG**R**G GGTCGTTCCGCCACTGGGAGGTGAAAGAGGGCAA
	AX87944147	5.45×10^{-5} (L)/ 2.78×10^{-5} (W)	CAGAGAGAGGGAGACCAGCGAGAGGGACACCAGAA **M**AAAAAGAAGGGAGGAAATTGAAGATGTTGCGGCGG
	AX87934338	7.30×10^{-5} (L)/ 6.41×10^{-5} (W)	ATTTAGCCACAGGGCCTATCCTCCCATCCTTAGAC**Y**GG CAGAGAGAGATGTAGGGAGACAAAGGCCTTTCA
	AX87992121	2.67×10^{-4} (L)/ 9.54×10^{-5} (W)	GAGCACTCCAACATTAATTTATTCAGGCATATTTA**M**CA GGACAAAGAGCAGAGGCCAGACCCTCCCGCCAC
	AX87943138	4.82×10^{-4} (L)/ 1.17×10^{-4} (W)	CACTTCAAGAGTTTACACGAAAAGTTGTTCTTATT**R**CG GAAATTGCCTATTGAGCTTTCCTATTCATGTAT
Chr. 20	AX87998237	5.70×10^{-2} (L)/ 4.85×10^{-2} (W)	GCAATGGAGGATGGAGATAATGGTTATGGAGATGG**Y**A TGGAGCTGGAGAAGGAACTGAGTCAGGAAGAGAG
	AX88289827	1.84×10^{-2} (L)	TGGAATTGCCCCCATACACTAATTACAAATGAAAG**M**G GTCTCTAATCTGGTGCTTGTCCCTTTGTAGTGTC
Top 0.5% in weight	AX87888225	9.96×10^{-5}	AATCTGACAGCGGTTCTTTCTTTTACCCAGTCGGA**S**AGG CCTCCTGGCTGGGGGTGCAAGGGCAGTTCACA
	AX88223695	1.24×10^{-4}	GCCCTATCAGAGAGAAGGGGGGGTGATGATAACAA**Y**G AGGTAGCTTTTTCAATCAACAATCACTCTTTTTA
	AX87959413	1.25×10^{-4}	CCCTTCATCGTATGGACTGACCATAAAAACCTGAC**Y**TA CATCCAGACCGCCAAACGTCTGAACTCCCGGCA
Top 0.5% in length	AX88141678	5.45×10^{-5}	AGTTTTGGCTGGGGTAAGTAAAGGATACTTTCCAA**R**TA TTTATCTTTTTTTAGAAGGCCTACTCTAATTTA
	AX87959512	9.13×10^{-5}	CCCTTTCAAGCACACCTTTCTCAATAACCTGTGGC**Y**AGT TATTCACAATTTTTGATGAAGAAATAAGGTTT
	AX88083269	1.02×10^{-4}	TTCCAGAAGATCCAAGAGCTGATAGATGACAAGCA**R**G CTCTGGTGTTTGTTCTCATCGATGAGGTAAAGCA

* The *p*-value was given in Tsai *et al.* [14]. W, body weight; L, body length. The shadow is used to distinguish different sources of markers utilized in this study. The bold letter are the single nucleotide polymorphism used in this study.

Supplementary Table 2. The marker ID, *p*-value estimated by the GWA analysis, putative gene and flanking sequence of two informative SNPs. The results were given in Tsai *et al.* [14].

Marker	Genotype	# No of Fish	Weight (g, SE)	Length (mm, SE)
	G/G	22	128.58 (5.20)	225.50 (3.48)
	A/G	240	115.26 (1.58)	216.93 (1.06)
AX88141678	A/A	362	109.16 (1.22)	211.82 (0.81)
		PVE	0.06	0.07
		p-value	4.12×10^{-4}	5.45×10^{-5}
	G/G	153	119.25 (2.11)	218.54 (1.42)
	A/G	355	110.98 (1.08)	213.69 (0.72)
AX88270804	A/A	116	106.25 (2.52)	210.50 (1.69)
		PVE	0.05	0.04
		p-value	1.32×10^{-3}	6.69×10^{-4}
